# Navigating PROSPERO4animals: 10 top tips for efficient pre-registration of your animal systematic review protocol

**DOI:** 10.1186/s12874-024-02146-0

**Published:** 2024-01-24

**Authors:** Alexandra Bannach-Brown, Torsten Rackoll, Nurcennet Kaynak, Natascha Drude, René Aquarius, Sofija Vojvodić, Mariana Abreu, Julia M. L. Menon, Kimberley E. Wever

**Affiliations:** 1 QUEST Center for Responsible Research, Berlin Institute of Health at Charité – Universitätsmedizin Berlin, Charitéplatz 1, 10117 Berlin, Germany; 2 Center for Stroke Research Berlin, Charité, Universitätsmedizin Berlin, Charitéplatz 1, 10117 Berlin, Germany; 3 Klinik Und Hochschulambulanz Für Neurologie, Charité, Universitätsmedizin Berlin, Charitéplatz 1, 10117 Berlin, Germany; 4 Berlin Institute of Health at Charité, Universitätsmedizin Berlin, Charitéplatz 1, 10117 Berlin, Germany; 5 Department of Neurosurgery, Nijmegen, Radboud University Medical Center, Internal Post Number 633, Geert Grooteplein-Zuid 30, 6525 GA Nijmegen, The Netherlands; 6 Instituto de Biofísica Carlos Chagas Filho, Universidade Federal Do Rio de Janeiro, Rio de Janeiro, RJ Brazil; 7 Brazilian Reproducibility Initiative in Preclinical Systematic Review and Meta- Analysis (BRISA) Collaboration, Rio de Janeiro, RJ Brazil; 8 Preclinicaltrials.Eu, Netherlands Heart Institute, Moreelspark 1, 3511 EP Utrecht, the Netherlands; 9 Department of Anesthesiology, Radboud Institute for Health Sciences, Radboud University Medical Center, Nijmegen, The Netherlands

**Keywords:** Systematic review, Preclinical research, Meta-research, Methodology, Pre-registration, Transparency

## Abstract

Systematic reviews are an essential tool in identifying knowledge gaps and synthesizing evidence from in vivo animal research to improve human health. The review process follows an explicit and systematic methodology to minimize bias, but is not immune to biases or methodological flaws. Pre-registering a systematic review protocol has several benefits, including avoiding unplanned duplication of reviews, reducing reporting biases, and providing structure throughout the review process. It also helps to align the opinions of review team members and can shield researchers from post-hoc critique. PROSPERO4animals is the international prospective register of systematic reviews (PROSPERO) for the preregistration of systematic review of animal studies. As administrators, here we provide 10 tips to facilitate pre-registration in PROSPERO4animals. These tips address common difficulties that both beginners and experienced researchers may face when pre-registering their systematic review protocols. This article aims to help authors write and register a detailed systematic review protocol on PROSPERO4animals.

**Supplementary Information:**

The online version contains supplementary material available at 10.1186/s12874-024-02146-0.

## Background

A systematic review aims to identify, appraise, and synthesize all empirical evidence relevant to a specific research question. First established in clinical research, this methodology is now increasingly used to synthesize evidence from in vivo animal research aimed at improving human health. There, this methodology is used to identify knowledge gaps, contribute to the implementation of the 3Rs, generate hypotheses for future research in animals or patients, and inform predictions about successful translation of preclinical findings to the clinical setting.

Systematic reviews follow explicit, systematic methodology, aimed at minimizing bias in the review process. However, this does not render them immune to biases or other methodological flaws. Therefore, recording the review question, methodology and analysis plan before observing the outcome is an essential step in producing high-quality reviews from which reliable conclusions can be drawn [1–3]. Pre-registering your systematic review protocol has many benefits to you as a researcher, as well as to the wider research community. It can help avoid unplanned duplication of systematic reviews, thereby reducing research waste, and can reduce reporting biases by enabling a comparison of the reported methods with what was initially planned [4]. Furthermore, completing a protocol helps you to plan your review in detail, thereby avoiding oversights regarding the tasks ahead and decisions to be made about the review methodology. It helps to align the opinions of all review team members and provides structure throughout the review process. Preregistration also prevents you from being taken hostage by your data when it comes to the synthesis stage, and can shield you from post-hoc critique, *e.g.,* in the peer-review process. It builds your reputation as a transparent researcher seeking high-quality results.

Despite all these benefits, you may find the prospect of pre-registration daunting, in terms of the expertise and time needed to complete a registration. The international prospective register of systematic reviews (PROSPERO; [5]) is an online platform that facilitates pre-registration of your systematic review protocol. It was launched in 2011, following the publication of the Preferred Reporting Items for Systematic reviews and Meta-Analyses (PRISMA) statement in 2010 [6] which advocates for registration of systematic review protocols, and in response to user demand. PROSPERO4animals (est. 2018) is the sub-section of this register dedicated to protocols of systematic reviews of animal studies. The history and scope of PROPSERO and PROSPERO4animals are described in more detail in Table 1 Box 1.

**Table 1:** Box 1—What PROSPERO4animals is and how to get there

The international prospective register of systematic reviews (PROSPERO; https://www.crd.york.ac.uk/prospero) is dedicated to the registration of systematic review protocols in nearly all fields of research where there is a health-related outcome. PROSPERO only accepted protocols for systematic reviews of human studies until 2018, when PROSPERO4animals, the section dedicated to systematic review protocols of animal studies, was launched. The scope of PROSPERO4animals is:
• **Systematic reviews of animal studies:** all submissions must aim to review evidence from animal studies. However, you may also include clinical, in vitro, or in silico studies if you plan to compare outcomes across evidence streams
• **Relevance to human health:** the review question must be relevant to human health. We presently do not have the scope to accept protocols of SRs investigating *e.g.,* veterinary questions or environmental exposure reviews only. For systematic review protocols outside of our scope, the database of veterinary systematic reviews (VetSRev; https://vetsrev.nottingham.ac.uk/), SYREAF (https://syreaf.org/), or Open Science Framework, are possible alternatives
• **Research purpose and a complete review team:** do not submit protocols for teaching or training purposes, including student projects. All submissions are manually assessed by the PROSPERO4animals admin team, and we do not have the capacity to handle submissions for teaching purposes or student projects
As of December 4th 2023, 2282 animal systematic review protocols have been registered. PROSPERO is funded by the National Institute for Health Research and managed by the York University Centre for Reviews and Dissemination. The administration of PROSPERO4animals is handled by the authors of this paper: a team of meta-researchers dedicated to improving the quality of animal systematic reviews. In addition to the 10 tips described here, our website https://www.crd.york.ac.uk/prospero/ provides extensive guidance and additional resources on performing systematic reviews of animal studies and how to complete your registration.

Over the past decade, PROSPERO administrators and advisory group members have continuously evaluated and improved the user experience, transparency and usefulness of the registry [7–9]. In recent years, we (as administrators) noticed that certain steps of the PROSPERO4animals registration process are sometimes difficult to navigate, for beginners and experienced researchers alike. To facilitate the process, we hereby provide 10 top tips to speedy pre-registration in PROSPERO4animals, the dedicated register for animal systematic review protocols.

### Our 10 top tips

1. Be honest and transparent.

Be open about the choices you are making in your systematic review methodology and make sure these choices support your research question. If this is the first time you are conducting a systematic review or meta-analysis, please include methodological experts (e.g., an information specialist and a meta-analysis expert) in your review team to improve the quality of your work. Ask a colleague or supervisor to read your protocol and discuss it before submission (a fillable template of the registration form is available in Appendix 1). In addition to PROSPERO, there are several other online resources to help you plan and perform your review:

- Radboudumc meta-research team: https://www.radboudumc.nl/meta-research-team
- CAMARADES systematic review resources: https://www.ed.ac.uk/clinical-brain-sciences/research/camarades/tools-resources/sr-animal-studies
- Preclinical Systematic Review Wiki: https://www.camarades.de/
- Preclinical Meta-Analysis Tutorial: https://camarades.shinyapps.io/R-MA-Tutorial/

Being honest and transparent also means that you should update your protocol if something changes along the way. You might find that an additional exclusion criterion is necessary to exclude irrelevant references, or you may realize during data extraction that you failed to prespecify a certain subgroup analysis. This is no problem as you can amend your registration at any moment in time. Amendments are a means to respond to unforeseeable events and a part of research if reported transparently. PROSPERO4animals offers version control: amendments will be time stamped, which enables readers to assess the differences between various versions of your protocol. Be brave and report what you plan at the beginning and change respective items, if necessary, at a later stage.

**Table:** 

*Example 1*
*Two researchers at the neurosurgery department want to investigate the efficacy of a certain endovascular device in murine aneurysm models. They register their protocol at PROSPERO4animals, but after running their search and piloting the selection process they realize that this device has also been tested in porcine models. They therefore broaden their research question and adjust their inclusion criteria to include porcine models, and record this decision by submitting an amendment to their PROSPERO4animals protocol*

2. Be realistic about your estimated completion date.

We are glad to see that you are enthusiastic and excited to complete your review. However, it is difficult even for a team of experts to complete a full systematic review and meta-analysis in under 3 months. It has been approximated that a systematic review of health interventions takes approximately 1 year [10], and many of the underlying estimates also hold true for animal study reviews. Be realistic when filling out the estimated completion date. If you find this difficult to estimate, the online tool PredicTER can be helpful to estimate how long your systematic review may take [11].

**Table:** 

*Example 2*
*Imagine that you and your colleague are two new PhD candidates and want to perform a systematic review to identify complications associated with radiation therapy in animal models for breast cancer. Your supervisor estimates it will take you 3 months to finish the review. However, after talking to a systematic review expert and using the online PredicTER *[11]* tool, you realize the review will take an estimated 12 months to complete. You discuss this with your supervisor, and you take the estimated 12 months into account when planning your other projects*

3. Do not start data extraction before registering your protocol.

To secure the benefits of prospective registration, PROSPERO does not accept protocols of reviews for which data extraction has started. Prospectively registering systematic review protocols helps to reduce potential reporting biases, avoid duplication of research efforts, and increases transparency [12]. If you became aware of the need for protocol registration at a later stage of your review, you can no longer register it at PROSPERO4animals. Alternatively, you can upload your protocol post-hoc to a repository such as Zenodo or Open Science Framework. Please refer to the PROSPERO website to review all criteria your protocol must adhere to, to be eligible for registration on PROSPERO.

**Table:** 

*Example 3*
*Two cardiologists have performed a systematic review on the effect of exercise on cardiac output in mouse models and want to submit their work to a journal. Reading though the ‘instructions for authors’ section, they find that the journal requires an *a priori* protocol registration accompanying any systematic review submission. One of them submits their protocol at PROSPERO4animals, but receives an email stating that the protocol has been rejected because data acquisition and analysis have already been performed. They must reconsider where to publish their work*

- 4. Use an appropriate review question structure.

Four common review question structures for animal systematic reviews are described below, along with examples published in the PROSPERO4animal register. Most animal systematic reviews aim to answer an interventional review question which follows the PICO (Population, Intervention, Control, Outcome) research question structure. If this applies to your review, please formulate your review question according to this format. This will help you fill out the sub-fields regarding inclusion criteria and exclusion criteria, which are also structured around the PICO format. If the PICO format is not suitable for your review, please explicitly state your research question structure. You may adapt the sub-fields for inclusion criteria and exclusion criteria accordingly, stating where certain sub-fields are not applicable.

**Table:** 

*Example 4*		
	**Structure**	**Example**
Intervention Questions		
PICO	For questions covering the effectiveness of an intervention, treatment, induction, etc Population (P) Intervention (I) Comparison (C) Outcome (O)	What is the effect of M. charantia preparations, versus control, on serum glucose level in animal models for type 2 diabetes mellitus? (see PROSPERO record CRD42019119181) P: animal models of type 2 diabetes mellitus I: M. charantia preparations C: treatment with vehicle, placebo, standard treatment or healthy animals O: serum glucose level
Exposure Questions		
PECO	For questions related to the effects of exposure to a factor / compound Population (P) Exposure (E) Comparison (C) Outcome (O)	Is there an association between the exposure to environmental endocrine disrupting chemicals and endometriosis in experimental mammalian animal models? (see PROSPERO record CRD42018102618) P: mammalian animal models E: environmental endocrine disrupting chemicals C: positive, negative or vehicle control O: endometriosis
Incidence / Prevalence Questions		
CoCoPop	For questions related to understanding the prevalence or incidence of a condition or problem Condition (Co) Context (Co) Population (Pop)	What is the true prevalence of anthroponotic and zoonotic soil-transmitted helminth infections in humans, canines and felines in Australian Indigenous communities and surrounds? (see PROSPERO record CRD42020165388) Condition (Co): helminth infections Context (Co): Australian Indigenous communities and surrounds Population (Pop): humans, canines and felines
Diagnostic Test Accuracy Questions		
PIRO	For questions related to the accuracy of a test at detecting a condition Population (P) Index Test (I) Reference or Comparator Test (R) Outcome (O)	What is the diagnostic performance of the loop-mediated isothermal amplification (LAMP) assay for canine visceral leishmaniasis when compared to current techniques? (see PROSPERO record CRD42022299722) Population (P): canines Index Test (I): Loop-mediated isothermal amplification (LAMP) assay Reference or Comparator Test (R): current techniques Outcome (O): canine visceral leishmaniasis

5. Use a comprehensive search strategy.

The comprehensive search is the foundation of your systematic review, so make sure you dedicate enough resources to this essential step. We highly recommend following the step-by-step guide to systematic searching for animal studies [1], taking tailored courses on systematic searching, and reaching out to your local librarian or information specialist for expert advice. Search in at least two bibliographic databases to retrieve all relevant evidence; commonly used databases include Medline, Embase and Web of Science, but your information specialist may suggest alternatives based on the topic of your review. Secondly, ensure that your search strings have high sensitivity by using free text terms (with appropriate field codes such as [tiab], which denotes searching in the title and abstract), as well as thesaurus terms (*i.e.,* Mesh or Emtree terms). You can assess whether your search strategy lives up to critical appraisal by evaluating it using *e.g.,* the Peer Review of Electronic Search Strategies.

(PRESS) guideline [13].

**Table:** 

*Example 5*
*Imagine that you and your colleague want to perform a systematic review on harmful effects of volatile anaesthetics on neonates in animal studies. You perform a search in PubMed using the terms ‘anaesthetic’, ‘volatile’, ‘neonate’ and ‘animal’. This search retrieves* ~ *1500 records, but many of those seem irrelevant to the review question. You decide to improve the search using the step-by-step guide* [1]*, and ask an information specialist at your university library for assistance. This helps you enhance the sensitivity and specificity of the search, by incorporating the correct field codes, synonyms, singular and plural spellings of terms, and using a search filter for animal studies. The information specialist also helps you translate the search to Embase. The improved searches now retrieve a much higher number of relevant references, as well as fewer irrelevant records*

- 6. Use complementary inclusion and exclusion criteria and prioritize them.

Inclusion and exclusion criteria determine the boundaries of the evidence base under review. Inclusion criteria describe the characteristics a study must have to be eligible for inclusion in your systematic review. Exclusion criteria describe which features make a study ineligible for your systematic review. Clearly defined and pre-specified eligibility criteria increase transparency and reduce the risk of bias in the record screening process [14]. Inclusion and exclusion criteria should be defined based on the key elements of the review question, *e.g.,* following the PICO structure (see Appendix 1).

Take particular care when defining the inclusion and exclusion criteria for study designs: this term is often mistakenly thought to refer to the publication type or population, while it should describe the design of the studies eligible for inclusion, such as randomized studies with separate control groups, cohort studies, observational studies etc.

In addition to defining them in detail, we strongly recommend prioritizing your exclusion criteria in a numbered list, ranking them from most easy to recognize to most difficult to recognize. This will help you save time making screening decisions and increase agreement between reviewers. Inclusion and exclusion criteria are often prioritized in the following order:

1. Publication type and publication limitations: is the article an ineligible publication type, e.g., a review, letter or editorial? Is the article published in the date range of interest?
2. Population: is the article an animal study? And if yes, is the animal used corresponding to the population of interest?
3. Intervention: does the article describe your intervention(s) of interest?
4. Outcomes: does the article investigate your outcome(s) of interest?
5. Study design: does the article describe an eligible study design?

**Table:** 

*Example 6* Examples of comprehensive, complementary inclusion and exclusion criteria for a PICO review question, and suggested prioritization			
**Element**	**Inclusion Criterion**	**Exclusion Criterion**	**Order**
Other	Full primary research articles	Not a full-length article, e.g., conference abstracts, reviews, or letters to the editor	1
Population	Pregnant mammals	Non-pregnant animals, non-mammals, humans, in vitro, ex vivo and in silico models or animals with co-morbidities	2
Intervention	Administration of stem cells, without any restriction on dose, duration of the intervention, route of administration, or frequency of administration	No stem cells administered, or stem cells combined with a co-intervention	3
Study Design	Studies with a separate control group	Studies without a control group, cross-over studies, or case studies / series	4
Control	Vehicle or placebo treatment	No treatment	5
Outcome	Spontaneous motor activity as measured using the open field test or cylinder test	Measurement of forced motor activity e.g., rotarod. Use of open field test for anxiety-like behaviour only	6

- 7. Be clear about the study characteristics and outcome data you aim to extract.

Pre-specifying which information you plan to extract from the included studies helps you extract and report this information in an unbiased manner [4]. The PROSPERO4animals protocol form therefore requires you to specify which study characteristics and outcome data you plan to extract (see Appendix 1). This section of the form follows the structure of the review question (e.g., PICO), but please do not repeat the inclusion criteria here. For example, for the Population, rather than stating “*animal models of type 2 diabetes mellitus*”, state which characteristics of the Population you wish to extract, *e.g.*, “*species, strain, sex and age*”. For outcome data, please record the type *(e.g.*, dichotomous or continuous) and all units of measurement you deem eligible for data synthesis (*e.g.*, “milligrams per decilitre”). If you anticipate units of measurement to vary widely between studies and you wish to include all of them, please state so, *e.g.*, *“outcomes are likely to be reported in various units of measurement, and continuous data in any unit of measurement will be extracted”.*

**Table:** 

*Example 7*
*Two researchers want to focus their systematic review on a new drug to treat atherosclerotic plaques in animal models. In their protocol they state that they will extract and synthesize data for two outcomes: 1) the number of plaques and 2) the plaque collagen content. Meta-analysis shows that the drug reduces the number of plaques, however, the plaque collagen content remains unchanged. Their co-author suggests including only the meta-analysis of the number of plaques in the manuscript for publication, to present a compelling message to the readers. However, the researchers refer to their review protocol, in which they stated that they will present both outcome measures, and convince their co-author to report all planned outcomes*

- 8. Familiarize yourself with critical appraisal tools for animal studies.

Critical appraisal of the included articles is a key step in the systematic review process, because the reliability of the conclusions of your review are highly dependent on the quality of the included evidence. Before submitting your protocol, familiarize yourself with the critical appraisal tools available to make sure the tool you choose is 1) suitable for animal studies and 2) fits the design(s) of the included studies. We strongly recommend assessing the internal validity (risk of bias) of the included studies using the SYstematic Review Centre for Laboratory animal Experimentation (SYRCLE) risk of bias tool [15], or a study quality assessment tool [16] tailored to animal studies (see Appendix 1). Read the tool's guidance beforehand so you can correctly record any additional criteria you want to assess, as well as the number of reviewers that will perform the assessment, and how discrepancies between their decisions will be resolved. Note that the Animal Research: Reporting of In Vivo Experiments (ARRIVE) guidelines [17] are a checklist for transparent reporting of in vivo experiments which cannot be used to assess internal validity or methodological quality.

**Table:** 

*Example 8*
*Imagine that you and your team are performing a systematic review on the effects of a new drug to reduce pain in animal models of rheumatoid arthritis. You are optimistic about the effects of the drug after outcome data extraction: it appears as if the positive effects are significant. However, your confidence in the evidence is dampened after performing the risk of bias assessment. Many studies do not report using measures to reduce bias, such as blinding or randomization. You are much more careful when interpreting results of your meta-analysis, and describe recommendations to improve the internal validity of future research in the discussion of your review*

- 9. Carefully consider which synthesis method fits your review.

Evidence synthesis is a key step in the systematic review process to provide insight into the evidence base as a whole, transcending the level of the individual study results. Please start this section of your protocol by describing which approach to data synthesis you intend to use: meta-analysis, or an alternative synthesis method (see Appendix 1). Both approaches require methodological expertise, which should be present in the review team before submitting the protocol.

For meta-analysis to be suitable, the studies should be similar enough regarding *e.g.*, the animal model used and the outcome measures you intend to combine. Secondly, enough studies need to have reported quantitative data for one of your review's outcomes. Because of the specific characteristics of animal study data (see tip 10), the power of meta-analysis rapidly decreases when only a small number of studies is included [18]. This holds true for the overall analysis and especially for subgroup analyses. To avoid uncertainty and potential bias at the analysis phase, the intention to use meta-analysis, the minimum number of studies required, and how you decide whether studies are similar enough needs to be planned and clearly stated in your protocol. Further, consider limiting the number of primary outcomes you pre-specify to reduce the type I error rate [18].

Although it may be tempting to view meta-analysis as a holy grail, please consider beforehand whether it will be appropriate to statistically combine the results of the included studies. More descriptive synthesis methods, such as vote counting based on the direction of effect, may be a better fit for your review. For descriptive synthesis, guidance tailored to animal studies is limited, but useful recommendations on preferred and inappropriate methods are provided by Cochrane [19] and the Joanna Briggs Institute [20].

**Table:** 

*Example 9*
*Two researchers are performing a systematic review to gauge the effects of probiotic use on the intestinal flora in animals, compared to a placebo treatment. They are aware that many studies have been published on the topic, but they anticipate that these will vary widely in terms of the species, treatment duration, outcomes measured, and the use of appropriate controls groups. Because of this heterogeneity, it does not seem sensible to statistically combine these studies in a meta-analysis. The researchers therefore decide to perform a narrative synthesis and describe this in their PROSPERO4animals protocol*

- 10. When you decide on meta-analysis, dive into the details.

If you decide that meta-analysis is sensible and feasible, plan each step of the meta-analysis in detail (see Appendix 1). First make sure you understand the basic underlying statistical principles [2]. Secondly, be aware that animal study data has unique characteristics which are very different from clinical study data, particularly the small sample size per study, the exploratory nature of the studies leading to high between-study heterogeneity, and the occurrence of multiple experimental comparisons per study. Guidelines for meta-analysis of clinical trials can therefore not be directly applied to animal study data and tailored methodology for meta-analysis of animal studies has been developed [2, 21].

Your protocol must include your planned choice of effect measure(s), meta-analysis model and heterogeneity statistics [2, 3, 18]. The effect measure used needs to be reported separately for each outcome you intend to analyse, since they depend on the nature of the outcome (continuous versus dichotomous) and the expected range in units of measurement. Regarding the effect models used to pool the data, the choice between a fixed effect and random effects model should be based on the statistical assumption underlying the data, not the amount of heterogeneity observed. In preclinical meta-analysis, it is highly recommended to use the random effects model, due to the underlying assumption that each study has a different “true effect” [18]. This between-study heterogeneity can be explored using subgroup analysis, which should be prespecified as much as possible. Consider a priori which study characteristics could contribute to differences in your effects of interest (e.g., whether the effect of the intervention differs between species, drug classes, etc.) and define in your protocol which sources of heterogeneity you plan to investigate.

**Table:** 

*Example 10*
*Imagine that you and your colleague are working on a systematic review to assess how model induction parameters affect the brain infarct volume in animal models of stroke. When planning your meta-analysis methodology, you agree that you will use data from the latest infarct volume measurement for each study. You also decide to use the standardized mean difference to account for differences in stroke volume between species, and plan to investigate heterogeneity using a meta-regression of the duration of middle cerebral artery occlusion. You record your plans in your review protocol accordingly*

### PROSPERO4animals registration process & our team

We are a team of enthusiastic volunteers with ample experience in performing systematic reviews of animal studies in a variety of fields, as well as developing systematic review methodology. We manually check each individual protocol submitted to PROSPERO4animals for eligibility and completeness (see Appendix 1 and the PROSPERO website for guidance), and put effort into writing tailored comments. Transparency is our guiding principle; responsibility for the relevance of the review topic and the quality of the planned methodology reside with you as the author, although we occasionally provide methodological advice to help you make the best out of your systematic review. Our comments may be the first external feedback you receive on your review protocol, and we hope you use them to enhance your research.

**Fig. 1:**
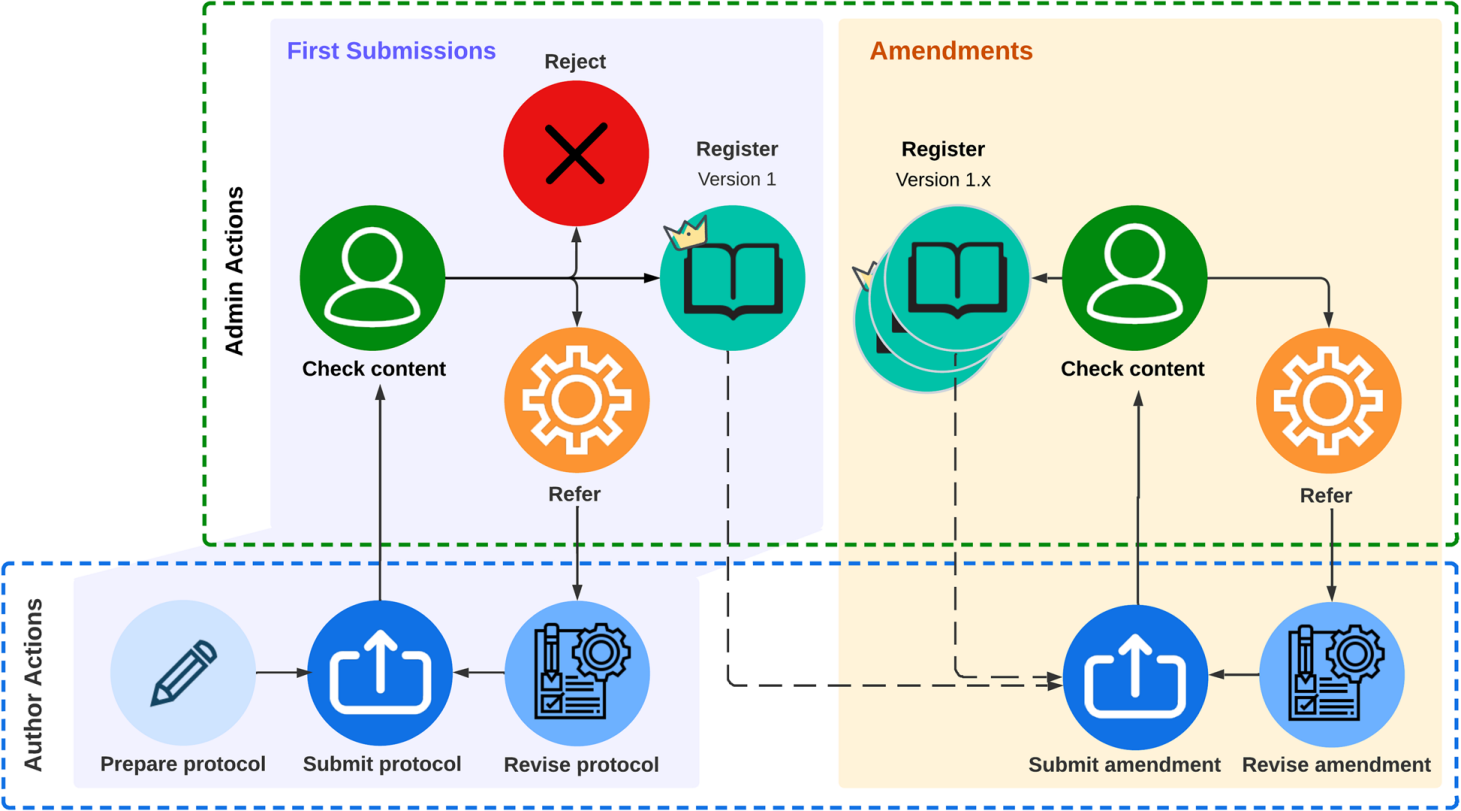
Steps in the registration process of PROSPERO4animals. After preparing their protocol, authors submit it to the admin team, who check the content and make the decision to reject it, register it as is, or refer it back to the author for revisions. After registration of the first version, authors may submit amendments to their protocol at any time. The admin team will check the content, after which the amendment will either be sent back to the authors for revisions, or be registered as a time-stamped, updated version of the protocol, supported by revision notes explaining the changes made

Figure 1 depicts the PROSPERO4animals registration process. We handle most of the new and revised submissions during our 1-h weekly video call, which allows us to discuss edge cases live where needed. Newly submitted protocols may be accepted as they are (when everything is in order), rejected (when the protocol is not eligible for registration in PROSPERO4animals) or, most often, referred back to the authors because the protocol is not yet complete or partly incorrect. When a referred protocol is updated and re-submitted by the author, we will re-assess it and decide again whether to accept, reject or refer it for a second revision. We usually strive for 1 revision round per protocol to keep timelines and workload manageable for ourselves and the authors. We aim to handle each protocol within 14 days of its submission, but this timeline may be exceeded, depending on the number of submission and the level of detail required for some protocols.

## Concluding remarks

Preregistration of a protocol for a systematic review is a valuable tool that can improve the transparency, rigor, and quality of the review process. It can also help authors avoid bias, increase accountability, and reduce publication bias. We therefore hope that you view preregistration at PROSPERO4animals as a method to add value to your planned review methodology, rather than as a box-ticking exercise. Writing and registering your PROSPERO4animals protocol will help you to identify possible hurdles in your planned methodology at the start of your systematic review process. For further methodological and educational resources on the planning and conduct of systematic reviews of animal studies, please refer to the references and links provided in Tip #1. By following these 10 tips, authors can navigate common difficulties in completing a registration in PROSPERO4animals, speed up the registration process, and improve the quality, transparency, and reproducibility of their systematic review. We wish you all the best for your future systematic review endeavours.

### Supplementary Information

**Additional file 1. **

## Data Availability

Not applicable.
