# Supplementary material for: Navigating PROSPERO4animals: 10 top tips for efficient pre-registration of your animal systematic review protocol
Source: BMC Med Res Methodol. 2024 Jan 24;24:20. doi: 10.1186/s12874-024-02146-0 (PMC10807142; doi:10.1186/s12874-024-02146-0)
Supplement: Supplementary file 1 — Additional file 1. [file 12874_2024_2146_MOESM1_ESM.docx]

**Appendix 1 to “Navigating PROSPERO4animals: 10 Top Tips for Efficient Pre-registration of your Animal Systematic Review Protocol” by Bannach-Brown et al.**

This appendix provides the PROSPERO4animals protocol template in an editable format, which authors can use to prepare their submission and discuss it with their colleagues. Items with an asterisk (*) are mandatory. The cursive text below each item name provides further instructions on how to complete the form.

| **Item #** | **Section/Subsection** | | | | **Enter your methods descriptions in this column** | | |
| --- | --- | --- | --- | --- | --- | --- | --- |
| **General Information** | | | | | | | |
| **1*** | Review Title  *In English, include intervention/exposure and health problem of interest* | | | |  | | |
| **2** | Original language title | | | |  | | |
| **3*** | Anticipated or actual start date | | | |  | | |
| **4*** | Anticipated completion date | | | |  | | |
| **5*** | Stage of review at time of this submission  *Indicate the stage of progress of the review by ticking the relevant Started and Completed boxes* | | Preliminary searches  Piloting of the study selection process  Formal screening  Data extraction  Risk of bias (quality) assessment  Data analysis | | | Started | Completed |
| **6*** | Named contact | | | |  | | |
| **7*** | Named contact email | | | |  | | |
| **8*** | Named contact address | | | |  | | |
| **9*** | Named contact phone number  *Do not forget international dialling code* | | | |  | | |
| **10*** | Organisational affiliation of the review | | | |  | | |
| **11*** | Review team members and their affiliations  *Fill this for each member involved in the review* | | | | Title:  First name:  Last name:  Organisational affiliation:  Email:  Country:  *[repeat for each author]* | | |
| **12*** | Funding sources/sponsors  *Add info, including grant number(s) if eligible* | | | |  | | |
| **13*** | Conflict of interest | | | | None  Yes  If yes, specify: | | |
| **14** | Collaborators  *List any individual who are working on the review but not listed as team members* | | | |  | | |
| **Context and searches** | | | | | | | |
| **15*** | Review question  *State the review questions, if eligible following a PICO or PO format* | | | |  | | |
|  | Context and rationale  *Brief description of the context, aim of the review, and relevance of the review for human health* | | | |  | | |
| **16*** | Searches  *Give details of the sources to be searched and any restrictions (e.g. language or publication period). Full search strategy is not required but can be added as a link or attachment* | | | |  | | |
| **17** | URL to search strategy  *It is also possible to upload the search in PDF format* | | | |  | | |
| **18*** | Human disease modelled  *State briefly the disease, condition or health care domain of interest* | | | |  | | |
| **Eligibility criteria of individual studies** | | | | | | | |
|  | *Make sure that all inclusion/exclusion criteria are complementary to each other and always write information for both inclusion and exclusion criteria. If one item does not apply to your review or if article will be selected regardless of that item, then write “none”.* | | | | | | |
| **19*** | Animal/population  *Give summary criteria for the animals being studied by the review, e.g. species, sex, details of disease model.* | | | | Inclusion criteria:  Exclusion criteria: | | |
| **20*** | Intervention(s), exposure(s)  *Give full and clear descriptions of the nature of the interventions or the exposures to be reviewed (e.g. dosage, timing, frequency).* | | | | Inclusion criteria:  Exclusion criteria: | | |
| **21*** | Comparator(s)/control  *Where relevant, give details of the type(s) of control interventions against which the experimental condition(s) will be compared (e.g. another intervention or a non-exposed control group).* | | | | Inclusion criteria:  Exclusion criteria: | | |
| **22*** | Study designs to be included  *Give details of the study designs eligible for inclusion in the review. If there are no restrictions on the types of study design eligible for inclusion, or certain study types are excluded, this should be stated.* | | | | Inclusion criteria:  Exclusion criteria: | | |
| **23*** | Other selection criteria or limitations applied  *Give details of any other inclusion and exclusion criteria, e.g. publication types (reviews, conference abstracts), publication date, or language restrictions.* | | | | Inclusion criteria:  Exclusion criteria: | | |
| **24*** | Outcome measure(s)  *Give detail of the outcome measures to be considered for inclusion in the review.* | | | | Inclusion criteria:  Exclusion criteria: | | |
| **Study selection and data extraction** | | | | | | | |
| **26*** | Procedure for study selection  *Give the procedure for selecting studies for the review, including the screening phases (title and/or title-abstract and/or full-text), the number of researchers involved, and how discrepancies will be resolved.* | | | |  | | |
|  | Prioritise the exclusion criteria  *Please sort the exclusion criteria defined in questions 19 to 24. If applicable, do so for each screening phase.* | | | | Selection phase:  1)  2)  3)  ….  Selection phase:  1)  2)  3)  … | | |
|  | Methods for data extraction  *Describe methods for data extraction, including the number of reviewers performing data extraction, extraction of data from text and/or graphs, whether and how authors of eligible studies will be contacted to provide missing or additional data, etc.* | | | |  | | |
|  | Data to be extracted: study design  *Specify the data to be extracted related to characteristics of the study design, e.g. controlled versus cross-over, number of experimental groups, etc.* | | | |  | | |
|  | Data to be extracted: animal model  *Specify the data to be extracted related to characteristics of the animal model, e.g. species, sex of the animals, etc.* | | | |  | | |
|  | Data to be extracted: intervention of interest  *Specify the data to be extracted related to characteristics of the intervention of interest, e.g. dose, timing, etc.* | | | |  | | |
|  | Data to be extracted: primary outcome(s)  *Define the primary outcome measure(s). For each outcome measure, specify in which format data will be extracted, including the eligible units of measurement, and data type (continuous/dichotomous). A description of any other manipulation or transformation of the extracted data that is planned may be included.* | | | |  | | |
|  | Data to be extracted: secondary outcome(s)  *Define the secondary outcome measure(s). For each outcome measure, specify in which format data will be extracted, including the eligible units of measurement, and data type (continuous/dichotomous). A description of any other manipulation or transformation of the extracted data that is planned may be included.* | | | |  | | |
|  | Data to be extracted: other  *Specify any other data or study characteristics to be extracted, e.g. bibliographical details, such as author, year and language.* | | | |  | | |
| **Risk of bias analysis and strategy for data synthesis** | | | | | | | |
| **27*** | Risk of bias and/or quality assessment  *State whether and how risk of bias and/or study quality will be assessed. Assessment tools specific for pre-clinical animal studies include SYRCLE’s risk of bias tool and the CAMARADES checklist for study quality.* | No risk of bias and/or quality assessment planned  By use of SYRCLE’s risk of bias tool  By use of SYRCLE’s risk of bias tool adapted as follows: *[specify]*  By use of the CAMARADES checklist for study quality  By use of the CAMARADES checklist for study quality adapted as follows: *[specify]*  Other criteria, namely: *[specify]* | | | | | |
|  | Method for risk of bias and/or quality assessment  *Give the procedure for the risk of bias and/or quality assessment, including the number of reviewers involved, their contribution, and how discrepancies will be resolved.* | | | |  | | |
| **28*** | Strategy for data synthesis  Planned approach  *For each outcome measure, specify whether a quantitative or narrative synthesis is planned and how this decision will be made.* | | | |  | | |
|  | If a meta-analysis is planned, please fill in the following: | | | |  | | |
|  | Effect measure  *For each outcome measure, specify the effect measure to be used (e.g. mean difference, odds ratio etc.).* | | | |  | | |
|  | Effect models  *For each outcome measure, specify the statistical model of analysis (e.g. random-effects or fixed-effect model).* | | | |  | | |
|  | Heterogeneity  *Specify the statistical methods to assess heterogeneity (e.g. I², Q). For further guidance, please refer to the introduction and practical guide to pre-clinical meta-analysis.* | | | |  | | |
|  | Other  *Specify other details of the meta-analysis methodology (e.g. correction for multiple testing, correction for multiple use of control group).* | | | |  | | |
| **29*** | Subgroup analyses  *Give any planned exploration of subgroups or subsets within the review. ‘None planned’ is a valid response if no subgroup analyses are planned.* | | | |  | | |
|  | Sensitivity  *For each outcome measure, specify any sensitivity analyses you propose to perform.* | | | |  | | |
|  | Publication bias  *Specify whether an assessment of publication bias is planned. If applicable, specify the method for assessment of publication bias.* | | | |  | | |
| **30*** | Review type | | | | Type of review  Animal model review  Experimental animal exposure review  Pre-clinical animal intervention review | | |
| **31** | Language | | | |  | | |
| **32*** | Country  *Select the country in which the review is being carried out from the drop down list. For multi-national collaborations select all the countries involved.* | | | |  | | |
| **33** | Other registration details  List other places where the systematic review protocol is registered. The name of the organisation and any unique identification number assigned to the review by that organisation should be included. | | | |  | | |
| **34** | Reference and/or URL for published protocol  *Give the citation and link for the published protocol, if there is one. Add web link to the published protocol.*  *Alternatively, upload your published protocol here in pdf format. Note that the upload will be publicly accessible.* | | | |  | | |
| **35** | Dissemination plans  *Give brief details of plans for communicating essential messages from the review to the appropriate audiences.* | | | |  | | |
| **36*** | Keywords  *Give words or phrases that best describe the review. Separate keywords with a semicolon or new line.* | | | |  | | |
| **37** | Details of any existing review of the same topic by the same authors  *Give details of earlier versions of the systematic review if an update of an existing review is being registered, including full bibliographic reference if possible.* | | | |  | | |
| **38*** | Current review status  *Review status should be updated when the review is completed and when it is published.* | | | Ongoing  Completed but not published  Completed and published  Completed, published and being updated , including Living Systematic Reviews  Discontinued | | | |
| **39** | Any additional information  *Provide any further information the review team consider relevant to the registration of the review.* | | | |  | | |
| **40** | Details of final report/publication(s) or preprints if available  *This field should be left empty until details of the completed review are available OR you have a link to a preprint. Give the full citation for the preprint or final report or publication of the systematic review.*  *Give the link to the published review or preprint.* | | | |  | | |
